# Effects of staged rehabilitation training on inflammatory factor levels and red blood cell distribution width followingcardiac valve replacement

**DOI:** 10.1186/s12872-024-03743-z

**Published:** 2024-03-13

**Authors:** Beibei Xing, Pujuan Liu

**Affiliations:** 1 Department of Emergency, The First Hospital of Hebei Medical University, Shijiazhuang, Hebei China; 2 Department of Cardiac Surgery, The First Hospital of Hebei Medical University, Shijiazhuang, Hebei China

**Keywords:** Staged rehabilitation training, Cardiac valve replacement, Inflammatory factor, Red blood cell distribution width

## Abstract

**Background:**

The current study was conducted aimed atexploring the effects of staged rehabilitation training on the levels of inflammatory factors and red blood cell distribution in patients who underwent cardiac valve replacement.

**Methods:**

A total of 140 patients who underwent cardiac valve replacement at The First Hospital of Hebei Medical University between April 2021 and November 2022 were included in this study. During the postoperative rehabilitation phase, the patients were randomly assigned to either the control group or the experimental group. The experimental group received staged rehabilitation training (*n* = 70), while the control group received conventional care and rehabilitation suggestions without specialized staged rehabilitation training (*n* = 70). Informed consent was obtained from all patients prior to theirinclusion in the study. Clinical data of the patients were collected andanalyzed. RDW was measured using an automated blood cell analyzer on postoperative day 1, 14, and 28. Levels ofTNF-α, IL-6 and CRP were measured using ELISA. Quality of life was evaluated usingthe WHOQOL-BREF questionnaire. The effects of postoperative rehabilitation were assessed using the 6MWD test. The occurrence of adverse events in the postoperative periodwas alsoanalyzed.

**Results:**

There were no significant differences in the general characteristics of the two groups of patients (*P* > 0.05). On the first day after surgery, no significant differences were seen in RDW between the two groups (*P* > 0.05). However, on the 14th and 28th day after surgery, the experimental group exhibited a significant reduction in RDW compared to the control group (*P* < 0.05). On the first day after surgery, the levels of serum TNF-α, IL-6 and CRP were comparable between the two groups (*P* > 0.05). However, on the 14th and the 28th after surgery, the experimental group showed evidently lower levels of TNF-α, IL-6 and CRP compared to the control group (*P* < 0.05). The experimental group demonstrated higher scores in the domains of physical health, psychological state, social relationships, and environment in the WHOQOL-BREF questionnaire compared to the control group (*P* < 0.05). Furthermore, the experimental group exhibited increased average,minimum,maximum walking distances in the6-minute walking test compared to the control group (*P* < 0.05). There were no significant differences in the incidence of postoperative adverse events between the two groups of patients (*P* > 0.05).

**Conclusion:**

Staged rehabilitation training exerteda positive effect on the levels of inflammatory factors and red blood cell distribution in patients following cardiac valve replacement. This type of rehabilitation training facilitated the patient’s recovery process by reducing the inflammatory response and improving the condition of red blood cells. Additionally, it enhanced the quality of life and rehabilitation outcomes for patients.

## Background

Heart valve replacement surgery is a common and important procedure for treating heart valve diseases [1]. Although surgery effectively improves valve function and enhances the quality of life, it remains an invasive treatment that necessitates a subsequent rehabilitation process to facilitate physical recovery and functional improvement [2]. In recent years, staged rehabilitation training has gained attention as a systematic rehabilitation strategy after heart valve replacement, incorporating comprehensive intervention measures such as sports training, psychological support, and education to facilitate gradual recovery of patient function and reduce the risk of complications [3–5]. However, limited research has been conducted on the impact of staged rehabilitation training on patients after heart valve replacement, particularly in relation to inflammatory factors and red blood cell distribution width (RDW). Inflammatory factors play a crucial role in the postoperative inflammatory response following heart valve replacement [6]. Surgical trauma and tissue injury trigger an inflammatory response, leading to the release of inflammatory factors [7]. Commonly studied inflammatory factors include TNF-α, IL-6,and CRP. Elevated levels of these factors have been found to correlate with the deterioration of postoperative complications. Additionally, RDW, an index reflecting the heterogeneity of red blood cell volume, is considered a biomarker of the inflammatory state [8, 9]. Inflammation can induce morphological and functional changesinred blood cells, thereby affecting the RDW value [10]. Therefore, monitoring postoperative RDW levels can provide important information about the inflammatory state and rehabilitation process [11]. Given this context, it is of great significance to investigate the effects of monthd rehabilitation training on inflammatory factors and RDW levels after heart valve replacement. Understanding the regulatory effects of staged rehabilitation training on these parameterscan offer ascientific basis and guidance for postoperative rehabilitation management. Thus, we aimed to explore the overall effects of staged rehabilitation training on inflammatory factors and RDW levels after heart valve replacement, providing new insightsand strategies for the rehabilitation of patients followingheart valve replacement.

## Methods

### Clinical background

A total of 140 patients were recruited from April 2021 to November 2022 from the cardiothoracic department of The First Hospital of Hebei Medical University. The study enrolled patients between theages of 53 and 70 who had undergoneheart valve replacement, with an average of 63.28 ± 6.44 years. Among the participants, there were 47 males and 93 females. For postoperative rehabilitation training, the patients were randomly divided into two groups: the control group and the experimental group. The experimental group, consisting of 70 patients, received staged rehabilitation training, while the the control group, also comprising 70 patients, received routine nursing and rehabilitation advice, but did not undergo specific staged rehabilitation training. All patients signed informed consent in accordance with the protocol before participating the current study.

### Inclusioncriteria

1. Patients between 18 and 70 years old.
2. Successful completion of heart valve replacement surgery with New York Cardiology Associationfunctional classification between grade II and III.
3. Absence of cognitive or nervous system disorders.
4. No active infection or inflammatory disease.
5. Voluntary participation and ability to engage in monthd rehabilitation training.
6. Signed informed consent form.
7. In addition, the subjects selected for this study were all undergoing mitral valve re-placement surgery.
8. All patients in this study did not receive medication or blood transfusion for hemoglobin levels.

### Exclusion criteria

1. 18–70 years old;
2. Having basic diseases such as severe heart disease, liver disease, and kidney disease;
3. Uncontrollable hypertension and diabetes patients;
4. Other comorbidities such as severe lung diseases and immune system diseases;
5. Patients who have previously undergone other cardiac surgeries or related treatments;
6. Those who have undergone valve replacement surgery and stent implantation surgery (mixed surgery);
7. Bone and joint diseases and neurological diseases that affect movement;
8. Do not agree to sign the informed consent form for the plan.

### Medical ethicalconsiderations

This study adhered to the Declaration of Helsinki and was approved by the Ethics Committee (full name: Ethics Committee of The First Hospital of Hebei Medical University). The authors declared that all methods were carried out in accordance with relevant guidelines and regulations. Written informed consent was obtained from all the participants.

The intervention lasted for a total of 3 months, and was implemented in the outpatient department every month. Postoperative recovery begins on the first day. Rehabilitation treatment with clinical intervention should be performed once a day. After discharge, the patient continued to receive rehabilitation treatment and went to the outpatient clinic for treatment. In the clinical rehabilitation stage, there is no training, only exercise. The training starts from the second month, including endurance training (on a treadmill or bicycle dynamometer), instrument resistance training, and general condition training. Each subject underwent the same strength training, aerobic training, and large muscle group training. Throughout the rehabilitation treatment process, the light load is set at 15RM+.

## Methods

### Postoperative rehabilitation training

(1) The control group patients received routine care and rehabilitation advice, including the process of adaptation. The control group patients also received routine rehabilitation treatment under strict care and monitoring, and received routine continuing care guidance and regular follow-up after discharge. The control group patients also received routine rehabilitation treatment under strict care and monitoring, including sputum training: instructing patients to cough gently several times, then perform inhalation movements, hold their breath for a period of time, and cough up phlegm; Routine rehabilitation training: Guide patients to perform limb flexion and extension exercises while lying in bed.

(2) Patients in the experimental group received staged rehabilitation training after the operation, including the active power training month, aerobic exercise training month, and large muscle group participation training month. In contrast, patients in the control group received routine rehabilitation guidance. Medical staff closely monitored and recorded any adverse events that occurred during the rehabilitation training, intervening and providing appropriate treatment as necessary.

(3) After surgery, all patients were admitted to the ICU and given ventilator assisted ventilation. Vascular active drugs such as dopamine and milrinone were administered intravenously, and standardized monitoring treatment was given after heart valve surgery. Routine administration of low molecular weight heparin combined with warfarin or simple use of warfarin anticoagulation, maintaining an internationally standardized ratio between 1.8 and 2.5.

(4) After the hospitalization and rehabilitation, the patient will rest at home and regularly go to the outpatient department for rehabilitation training.

### Red blood cell distribution width detection

RDW was measured on the 1st, 14th and 28th days after the operation using automatic blood cell analyzer. Firstly, a 2 mL sample of anticoagulated blood was collected from the patients’ veins and transferred to atest tube for analysis. The automatic blood cell analyzer was calibrated according to the instrument’srequirements to ensure accurate blood cell count and parameter measurement. The test tube was then placed intheanalyzer, which calculated RDW and reportedits value as a percentage.

### ELISA detection

Patients were instructedto fast for 10 h prior to venous blood collection. The levels of inflammatory factors, including TNF-α, IL-6, and CRP, were measured using the enzyme-linked immunosorbent assay (ELISA) method.

### Six-minute walking distance test

The 6-minute walking distance (6MWD) test was carried out in a flat and enclosed hospital corridor,with the walking distance being recorded within 6 min. The purpose and process of the tests were explained to the patients by medical staff, ensuring their understanding and confirmingtheir physical suitability for walking activities. During the test, the medical staff recorded the start time of the patient’s walk and encouragedthem to maintain theirnormal walking speed. The distance covered within 6 min was monitored, and the recording ceased at the end of the allotted time. Patients were allowed moderate rest during the test but were advised against engaging in strenuous exercise. The average, minimum, and maximum distanceswalked by each patient during the 6-minute period were recorded.

### WHOQOL-BREF scale score

WHOQOL-BREF scale was used to assess the quality of life of the patients in our study. This widely utilized assessment tool consists of four domains: physical health, psychological state, social relationships, and environment, encompassing a total of 26 questions. Each question has a designed scoring range, and patient’s selected answercorresponds to a score within that range. Physical health: This domainevaluates the physiological function and health status,comprising 4 questions. Each question is scored on a scale of 1–5. Psychological state: This domain evaluates patients’ psychological state, emotions, and cognitive function, consisting of 6 questions. And each question is scored on a scale of 1–5. Social relationships: This domain assesses patients’ social interactions, interpersonal relationships, and social support, including3 questions, with each question being scored on a scale of1-5. Environment: This domain evaluates patients’ living environment, economic situation, personal safety, and access to social services,comprising 8 questions, with each question being scored on a scale of1-5. The score foreach domain was calculated as the average score of the respective questions. The overall quality of life score was determined as the average score across allfour domains.

### Statistical analysis

All statistical analyses were doneusing SPSS version 26.0 statistical analysis software (SPSS Inc, Chicago, Illinois, USA). The distribution of data in this study follows a normal distribution. Continuous variables were compared using the unpaired student’s t-test, while the chi-square test was employed to assess the significance of differences in relevant data. A *p*-value less than 0.05 was considered statistically significant.

## Results

### Comparison of general characteristics

According to the clinical data of the patients, there were 25 males and 45 females in the control group, with an average age of 64.68 ± 5.21 years, a BMI of 23.26 ± 2.05 kg/m^2^, systolic blood pressure of 124.36 ± 11.69mmHg, diastolic blood pressure of 77.42 ± 8.36mmHg, heart rate of 68.34 ± 6.29, and incidence of 24 cases of hypertension, 13 cases of diabetes, and 17 cases with a history of smoking and drinking. The experimental group consisted of 22 male and 48 female patients, with an average age of 62.38 ± 6.68 years, a BMI of 23.47 ± 2.63 kg/m^2^, systolic blood pressure of 127.82 ± 12.49mmHg, diastolic blood pressure of 75.39 ± 7.24mmHg, heart rate of 67.31 ± 7.57, and a incidence of 26 cases of hypertension and 11 cases of diabetes. There wereno significant differencesin the general characteristics between the two groups (*P* > 0.05). (Table 1)

**Table 1:** Comparison of general characteristics

Parameter	Control group (*n* = 70)	Experimental group (*n* = 70)	T value /χ^2^ value	*P* value
Gender (male: female)	25: 45	twelve to eleven p.m.	1.004	0.325
Age (years)	64.68 ± 5.21	62.38 ± 6.68	0.945	0.115
BMI(kg/ m^2^)	23.26 ± 2.05	23.47 ± 2.63	2.557	0.546
Systolic pressure	124.36 ± 11.69	127.82 ± 12.49	4.682	0.662
Diastolic pressure	77.42 ± 8.36	75.39 ± 7.24	2.947	0.215
Heart rate	68.34 ± 6.29	67.31 ± 7.57	0.325	0.496
Hypertension	24(34.28%)	26(37.14%)	1.558	0.283
Diabetes	13(18.57%)	11(15.71%)	3.429	0.117
Smoking and drinking	17(24.28%)	20(28.57%)	3.801	0.406
Duration of antihypertensive medication use (years)	11.23 ± 3.21	12.85 ± 3.36	2.602	0.235

### Measurement of RDW after the operation

RDW was measured usingan automatic blood cell analyzer on the 1st, 14th, and 28th days after the operation. No significantdifference was seen between the two groups on the 1st day after the operation (*P* > 0.05). On the 14th day and 28th day after the operation, however, the RDW inthe experimental group was lower compared to thecontrol group (*P* < 0.05). (Table 2)

**Table 2:** Comparison of postoperative RDW of the two groups ($\bar x \pm s$,%)

Groups	The first day after operation	14th day after operation	The 28th day after operation
Control group (*n* = 70)	18.35 ± 0.64	17.21 ± 0.52	15.47 ± 0.36
Experimental group (*n* = 70)	18.42 ± 0.71	15.49 ± 0.38	12.36 ± 0.15
*T value*	2.168	11.402	19.533
*P value*	0.285	0.025	0.016

### Postoperative inflammatory factor levels

There were no significant differences in the serum levels of TNF-α, IL-6, and CRP between the two groups on the first day after the operation (*P* > 0.05). However, on the 14th and 28th days after the operation, the levels of TNF-α, IL-6, and CRP in the experimental group were remarkably lower than those in the control group (*P* < 0.05). (Table 3)

**Table 3:** Postoperative inflammatory factor levels of the two groups ($\bar x \pm s$, %)

Parameter	Time point	Control group (*n* = 70)	Experimental group (*n* = 70)	T value /χ^2^ value	*P* value
TNF-α(pg/ml)	The first day after operation	48.32 ± 3.78	46.28 ± 4.66	1.026	0.362
	14th day after operation	33.68 ± 2.49	25.53 ± 3.12	12.987	0.001
	The 28th day after operation	26.85 ± 1.50	20.53 ± 1.37	6.712	0.001
IL-6(pg/ml)	The first day after operation	65.77 ± 5.32	63.47 ± 5.17	1.025	0.563
	14th day after operation	53.24 ± 3.88	47.33 ± 4.29	15.367	0.001
	The 28th day after operation	38.19 ± 2.41	31.62 ± 2.38	7.115	0.001
CRP(mg/L )	The first day after operation	28.36 ± 3.19	29.41 ± 3.58	1.209	0.159
	14th day after operation	18.44 ± 1.25	12.65 ± 1.08	6.527	0.001
	The 28th day after operation	13.65 ± 1.05	8.63 ± 1.01	8.559	0.001

### WHOQOL-BREF scale score

The scores for physical health, psychological state, social relationships, and environment in the experimental group were evidently higher than those in the control group (*P* < 0.05), indicating that patients in the experimental group had a better quality of life. (Table 4)

**Table 4:** WHOQOL-BREF scale score of the two groups ($\bar x \pm s$)

Group	Physical health	Psychological state	Social relationships	Environment
Control group (*n* = 70)	3.64 ± 0.25	3.85 ± 0.34	3.15 ± 0.15	3.72 ± 0.24
Experimental group (*n* = 70)	4.18 ± 0.36	4.35 ± 0.40	3.86 ± 0.21	4.25 ± 0.31
*T value*	11.426	17.301	12.449	9.574
*P value*	0.012	0.004	0.025	0.006

### Comparison of 6MWD test

**Fig. 1:**
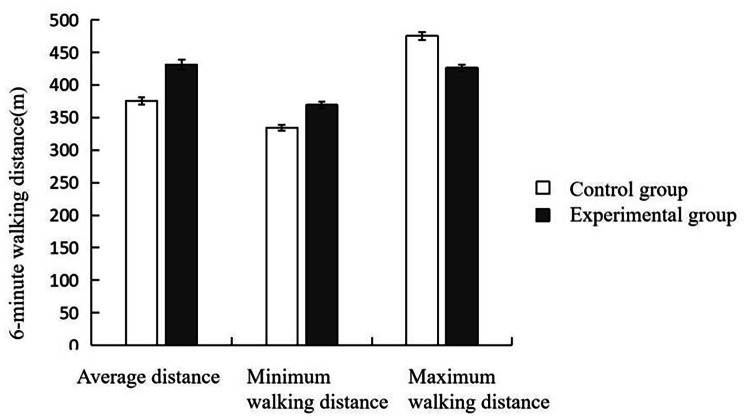
6-minute walking distance

By comparing the postoperative rehabilitation effects using6MWD test, it was found that the average, minimum, and maximum walking distance inthe experimental group were significantly increased compared to the control group (*P* < 0.05). This suggested that patients in the experimental group had improved exercise ability and cardiopulmonary function. (Fig. 1; Table 5)

**Table 5:** Comparison of 6MWD test ($\bar x \pm s$, m)

Group	Mean distance	Minimum walking distance	Maximum walking distance
Control group (*n* = 70)	375.62 ± 5.49	334.16 ± 4.29	475.36 ± 6.27
Experimental group (*n* = 70)	431.27 ± 7.38	369.45 ± 5.12	426.41 ± 5.37
*T value*	11.682	9.205	14.316
*P value*	0.012	0.022	0.006

### Statistics of postoperative adverse events

**Fig. 2:**
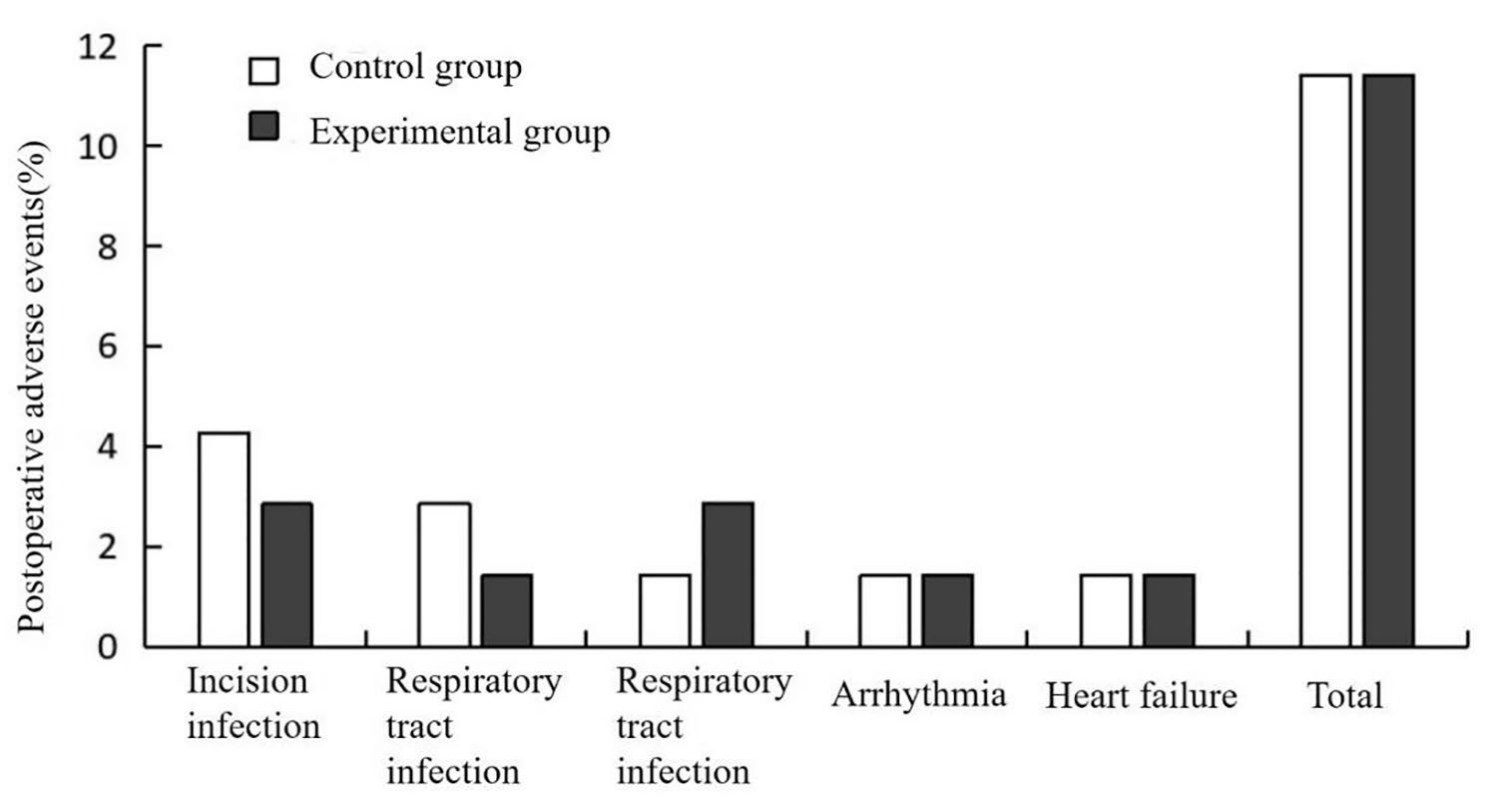
Postoperative adverse events

In the control group, there were 3 cases of incision infection, 2 cases of respiratory tract infection, and 3 cases of adverse vascular events, including 1 case of frequent heart rate, 1 case of myocardial infarction, and 1 case of heart failure. The experimental group, on the other hand,had 2 cases of incision infection, 1 case of respiratory infection, and 4 cases of cardiovascular events, including 2 cases of arrhythmia and 1 case of myocardial infarction with associated heart failure. There was no significant differencein the incidence of postoperative adverse events between the two groups (*P* > 0.05). (Fig. 2; Table 6)

**Table 6:** Statistics of postoperative adverse events ($\bar x \pm s$, m)

Group	Incision infection	Respiratory tract infection	Arrhythmia	Weak heart	cardiac infarction	Total (%)
Control group (*n* = 70)	3(4.28%)	2(2.85%)	1(1.43%)	1(1.43%)	1(1.43%)	8(11.42%)
Experimental group (*n* = 70)	2(2.85%)	1(1.43%)	2(2.86%)	1(1.43%)	1(1.43%)	7(11.42%)
*T value*	2.017	1.772	3.419	0.893	1.225	3.843
*P value*	0.403	0.215	0.568	0.491	0.152	0.264

## Discussion

Heart valve replacement surgery is a commonly used surgical intervention for the treatment of heart valve diseases. Despite being an invasive procedure, surgery alone is insufficient, and patients require a structured rehabilitation process post-surgery to facilitate prompt recovery of functions,alleviate symptoms, and minimize the risk of complications.

Staged rehabilitation training plays a pivotalrole in the postoperative rehabilitation of heart valvepatients [12, 13]. By implementing atargeted rehabilitation plan and comprehensive rehabilitation measures, staged rehabilitation train ingaids patients ingradually restoring heart function, enhancing physical activity capacity, and promoting holistic physical and mental rehabilitation [14]. First, staged rehabilitation training contributes to the restoration of heart function. Heart valve diseases often result in impaired heart function, necessitating adaptation to new valves and changes in heart function after surgery [15]. Rehabilitation training involves progressive increments in aerobic exercise, strength training, and cardiac monitoring, all of which assist in strengthening cardiac muscles, improving cardiac contractility and output, and enhancing overall heart function [16, 17]. Secondly, staged rehabilitation training facilitates improvements in patients’ physical activity capabilities [18]. Patients after surgery often encounter issues such as physical decline, muscle weakness, and fatigue, which hindertheir daily activitiesand work performance [19]. Rehabilitation training aidspatients ingradually improving physical strength, endurance, muscle strength, and overall physical function by gradually intensifying exercise intensity and frequency. As a result,patients can actively participate in social activities, work, and daily life. Additionally, staged rehabilitation training greatly contributes to patients’ psychological rehabilitation. Heart valve replacement surgery can negatively impactpatients’ psychological and emotional well-being, leading to anxiety, depression, and psychological stress [20, 21]. Rehabilitation training assists patients in managing postoperative psychological stress, bolstering psychological resilience, self-confidence, and improving mental health through psychological support, education, and behavioral interventions [22]. Staged rehabilitation training also plays a vital role in comprehensive patient rehabilitation. By providing comprehensive rehabilitation services, including psychological support, social interaction, and vocational rehabilitation, monthd rehabilitation training facilitates patients’ successful reintegration into society, restoration of work capabilities, and improvement of their overall quality of life [23, 24]. Consequently, it is crucial to recognize the significance of implementing staged rehabilitation training following heart valve surgery and strengthen the delivery of rehabilitation services in clinical practice. Future research endeavors should further explore the effects of different rehabilitation strategies and optimize rehabilitation programs to better addressthe diverse rehabilitation requirementsof patients [25].

The purpose of this study was to investigate the impact of staged rehabilitation training on the levels of inflammatory factors and RDW following heart valve replacement. Through a comparative analysisof 140 patients who underwentheart valve replacement, we observed significant improvementsin postoperative RDW and decreased levels of serum inflammatory factors (TNF-α, IL-6, and CRP) in the experimental group receiving staged rehabilitation training compared to the control group. Furthermore, patients in the experimental group exhibited enhanced quality of life of in the domains of physical health, psychological state, social relationships, and environment, with evident advantages observed in the 6MWD test.

In ourstudy, we noteda substantial decrease in RDW levelson the 14th and 28th day after the operation inthe experimental group,suggesting a positive influence of staged rehabilitation training on the overall rehabilitation process of patients. RDW serves asan indicator reflecting the size and variation of red blood cells, and elevated RDW levelsareoften associated with inflammatory reactions and the progression of cardiovascular diseases. Hence, the observed reduction in RDW in the experimental group implied that staged rehabilitation training may contribute to the mitigation of inflammatory reactions, alleviation of cardiovascular pressure, and improvement inred blood cell distribution characteristics. In addition, we also observed a downward trend in the levels of inflammatory factors in the experimental group compared to the control group. TNF-α, IL-6, and CRP are commonly employed inflammatory markers, and their elevated levels are typically linked to inflammatory reactions and tissue damage. The decrease inpostoperative TNF-α, IL-6, and CRP levels in the experimental group may indicated that staged rehabilitation training may bebeneficial ininhibiting the occurrence and progressionof inflammatory reactions. This effect mightbe achieved by enhancing patients’immune function, promoting blood circulation, and improving oxygenation. This study also revealeda significant improvement inthe quality of life of patients in the experimental group across multiple domains. Heart valve replacement surgery may negatively impact patients’ quality of life, and staged rehabilitation training provided more comprehensive rehabilitation care by facilitatingphysical function recovery, providing psychological support, and fostering social interaction. As a result, the experimental group exhibited notable enhancements in scores related to physical health, psychological state, social relationships and environment, indicating that staged rehabilitation training played a positive role in improving overall quality of life. In evaluating the rehabilitation effect, the study conductedan objective comparison through a 6MWD test. The experimental group demonstrated advantages in terms of average, minimum, and maximum walking distance of the 6-minute test, indicative of improved physical endurance, mobility, and rehabilitation outcomes resulting from staged rehabilitation training. However, despitethe positive effects observed in the abovementioned indicators, it should benoted that there was no significant difference in the incidence of postoperative adverse events between the two groups. This could be attributed to factors such as the small sample size, limitations in the research design, and the influence of other variables. Therefore, future research should aim to increase the sample size and further evaluate the influence of different rehabilitation strategies on the incidence of adverse events.

In summary, staged rehabilitation training demonstrated positive effects on the levels of inflammatory factors and RDW in patients following heart valve replacement surgery. Rehabilitation training facilitated the rehabilitation process by reducing inflammatory reactions and improving the conditionof red blood cells. Moreover, it enhanced patients’quality of life and rehabilitation outcomes. These findings emphasized the importance of staged rehabilitation training after heart valve replacement surgery and offered valuable guidance and reference for clinical practice. Future research should continue toexplore the specific content and duration of rehabilitation training in order to further optimize the rehabilitation strategy for patients undergoing heart valve replacement.

## Data Availability

The datasets analyzed during the current study are not publicly available due to the personal privacy but are available from the corresponding author on reasonable request.

## Abbreviations

RDW: Red blood cell distribution width
ELISA: Enzyme-linked immunosorbent assay
6MWD: 6-minute walking distance
